# Species- and Age/Generation-Dependent Adherence of *Bifidobacterium bifidum* to Human Intestinal Mucus In Vitro

**DOI:** 10.3390/microorganisms9030542

**Published:** 2021-03-05

**Authors:** Gaku Harata, Kazutoyo Yoda, Ruipeng Wang, Kenji Miyazawa, Masayuki Sato, Fang He, Akihito Endo

**Affiliations:** 1 Takanashi Milk Products Co., Ltd., Honjukucho-5, Asahi-ku, Yokohama, Kanagawa 241-0021, Japan; k-yoda@takanashi-milk.co.jp (K.Y.); z-oh@takanashi-milk.co.jp (R.W.); ke-miyazawa@takanashi-milk.co.jp (K.M.); m-satoh@takanashi-milk.co.jp (M.S.); ka-hou@takanashi-milk.co.jp (F.H.); 2 Department of Food, Aroma and Cosmetic Chemistry, Faculty of Bioindustry, Tokyo University of Agriculture, 196 Yasaka, Abashiri, Hokkaido 099-2493, Japan; a3endou@nodai.ac.jp

**Keywords:** *Bifidobacterium bifidum*, *B. bifidum* TMC3115, adhesion, mucus, *Lacticaseibacillus rhamnosus* GG, age effect

## Abstract

Adhesion to intestinal mucus is the first event in the process by which intestinal microbes colonize the intestine. It plays a critical role in the initiation of interactions between gut microbes and host animals. Despite the importance, the adhesion properties of probiotics are generally characterized using porcine mucin; adhesion to human mucus has been poorly characterized. In the present study, human intestinal mucus samples were isolated from 114 fecal samples collected from healthy infants and adults. In initial screening, four out of the 13 beneficial microbes tested, including the type strain of *Bifidobacterium bifidum, B. bifidum* TMC3115, *Lacticaseibacillus rhamnosus* GG, and *Bifidobacterium animalis* subsp. *lactis* Bb12, showed strong adhesion abilities to human mucus. The type strain of *B. bifidum* and TMC3115 adhered more strongly to neonatal and infant mucus than to adult mucus, while *L. rhamnosus* GG and *B. lactis* Bb12 adhered more strongly to adult mucus than to infant mucus. Similar results were obtained for ten additional strains of *B. bifidum*. In conclusion, age/generation-related differences were observed in the adhesion properties of *B. bifidum* and other strains. A deeper symbiotic relationship may exist between infants, particularly neonates, and *B. bifidum* based on its enhanced adhesion to neonatal intestinal mucus.

## 1. Introduction

Bifidobacteria are one of the main groups of the human intestinal microbiota and are predominant in infants [1,2]. Accumulating evidence has indicated that bifidobacteria play a critical role in human health and well-being [3,4]. Quantitative and qualitative abnormalities in bifidobacteria have been reported in various infections and physiological disorders. For example, low numbers of intestinal bifidobacteria would be an important aspect of the aberrant intestinal microbiota in allergic infants, and low numbers of *Bifidobacterium* have been detected before the onset of allergies [5,6]. Recent evidence supports previous findings showing that infants with allergies in Western countries may be less frequently colonized by infant-type *Bifidobacterium* species, such as *Bifidobacterium longum* subsp. *infantis*, *B. longum* subsp. *longum*, *B. bifidum,* and *B. breve*, and more frequently colonized by adult-type *Bifidobacterium*, including *B. catenulatum* and *B. adolescentis,* than infants without allergies [7,8,9,10]. Therefore, the gut *Bifidobacterium* population is a prospective target in the management of allergic diseases in infants. 

Human intestinal epithelial cells are covered by a thick mucus layer. Mucus is the initial surface that ingested microorganisms are confronted with in the human gut and is regarded as an important site for bacterial adhesion and colonization [11]. Mucus is continually subjected to degradation by gut microbes; in contrast, new mucin glycoproteins (the major components of mucus) are constantly secreted. Adhesion to intestinal mucus is the first event in the process by which intestinal microbes colonize the intestine and it plays a critical role in the initiation of interactions between intestinal microbes and host animals [11]. Based on these findings, an in vitro evaluation of bacterial adhesion to human intestinal mucus provides a good model for studying the ability of probiotics to adhere to intestinal surfaces. A number of studies have investigated the adhesion of bifidobacteria and lactic acid bacteria to epithelial cells using Caco2 and HT29 carcinoma cell lines and mucin originating from the porcine stomach [12,13,14]; however, limited information is currently available on adhesion to human mucus by these microbes. Moreover, few studies have characterized the adhesion properties of microbes to human mucus originating from different generations. 

Therefore, the present study was conducted to evaluate the adhesion properties of bifidobacteria to human intestinal mucus. Mucus originating from different ages or generations were included in the present study to characterize possible age/generation-dependent adhesion properties in the microbes. 

## 2. Materials and Methods

### 2.1. Bacterial Strains

Eight type strains of *Bifidobacterium,* including *B. adolescentis* JCM 2701 ^T^ (ADO), *B. angulatum* ATTC 27678 ^T^ (ANG), *B. longum* subsp. *infantis* JCM 1222 ^T^ (INF), *B. pseudocatenulatum* JCM 1200 ^T^ (PSE), *B. bifidum* JCM 1255 ^T^ (BIF), *B. breve* JCM 1192 ^T^ (BRE), *B. catenulatum* JCM 1194 ^T^ (CAT), and *B. longum* subsp. *longum* JCM 1217 ^T^ (LON), and *Akkermansia muciniphila* JCM 30893 (AKK) were obtained from the Japan Collection of Microorganisms (JCM; RIKEN Bioresource Center, Japan). *Lacticaseibacillus rhamnosus* GG (LGG) and *Bifidobacterium animalis* subsp. *lactis* Bb12 (Bb12) were supplied by Chr. Hansen (Hørsholm, Denmark). *B*. *bifidum* TMC3115 (TMC3115) was originally isolated from healthy infant stools and *L. rhamnosus* LA2 (LA2) from traditional fermented food. 

Ten additional strains of *B. bifidum* (TMC3103, TMC3104, TMC3108, TMC3110, TMC3112, TMC3116, TMC3119, TMC3120, TMC3121 and TMC3122) isolated from healthy infants [14] were also included in the present study. TMC strains belong to the Microbiological Collection of Takanashi (TMC) at the Technical Research Laboratory of Takanashi Milk Products Co., Ltd. (Yokohama, Japan).

De Man-Rogosa-Sharpe medium (Becton Dickinson, Sparks, MD, USA) was used to culture lactobacilli and bifidobacteria at 37 °C for 18 h, and *A. muciniphila* was routinely grown in GAM medium (Nissui Pharmaceutical, Tokyo, Japan). Cultures were incubated anaerobically at 37 °C for 18 h using the AnaeroPack system (Mitsubishi Gas Chemical Co., Tokyo, Japan), and growth was monitored by measuring O.D._600nm_ using the DU800 spectrophotometer (Beckman Coulter, Tokyo, Japan).

### 2.2. Isolation of Fecal Mucus

Volunteers were within 5 days old (*n* = 4), 1 month old (*n* = 9), 4 months old (*n* = 8), 6 months old (*n* = 11), 9 months old (*n* = 12), 1 year old (*n* = 12), 1 and a half years old (*n* = 10), 3 years old (*n* = 6), 20–29 years old (*n* = 9), 30–39 years old (*n* = 9), 40–48 years old (*n* = 13), and 50–59 years old (*n* = 11). Collected fecal samples were kept at −80 °C until used.

Fecal mucus was isolated using the same methods as those described in a previous study [15]. Briefly, a 2-g fecal sample was added to 8 mL of PBS and mixed for 2 h at 4 °C using an “end-over-end” mixer. After centrifugation at 10,000× *g* for 30 min at 4 °C, the supernatant was obtained and centrifuged at 15,000× *g* for 30 min at 4 °C to remove any particulate matter. The supernatant was mixed with 10 mL of ice-cold ethanol and then centrifuged at 8000× *g* for 20 min at 4 °C to obtain sediment. The sediment was dissolved in 10 mL of ice-cold milli-Q water. It was frozen at −70 °C overnight and lyophilized. The dried matter obtained was used as fecal mucus in the present study.

### 2.3. Mucus Binding Assay

Bacterial adhesion to mucus was determined according to a previously described procedure [14]. Briefly, human intestinal mucus extract and porcine mucin (porcine stomach, Sigma-Aldrich, St. Louis, MO, US) were passively immobilized on 96-well MaxiSorp plates (Nalge Nunc, Rochester, NY, US) by 50 mM carbonate/bicarbonate buffer at 4 °C overnight. The wells were washed three times with PBS and blocked for 1 h with PBS plus 1% Tween 20. Overnight cultured bacterial cells were adjusted to O.D._600 nm_ of 0.6 ± 0.03 (approximately 10^8^–10^9^ CFU/mL) with PBS. A 100 μL aliquot of bacterial cells was added to each well of the plates, which were then incubated at 37 °C for 2 h. Non-adhered bacterial cells were removed by washing three times with PBS plus 0.05% Tween 20, and plates were dried at 55 °C. Adhered cells were stained with crystal violet (1 mg/mL) for 45 min. After six washes with PBS, the colorant was liberated with 50 mM citrate buffer for 45 min and absorbance at 560 nm was determined using an ARVO™ MX plate reader (PerkinElmer Japan, Kanagawa, Japan). Adjusted bacterial cells without mucus were run as controls in all experiments. The human mucus binding assay was performed for each fecal mucus sample, and the porcine mucus binding assay was conducted in a quintuple well in triplicate using cells from independent cultures.

### 2.4. Statistical Analysis

Statistical analyses were performed using JMP version 10.0.0 (SAS. Institute Japan, Tokyo, Japan) software. Data were expressed as the mean ± the standard deviation for each sample. Differences between means were tested for significance using a one-way ANOVA followed by the Tukey Kramer or Kruskal–Wallis and Steel–Dwass tests. A *p*-value ≤ 0.05 was considered to be significant.

## 3. Results

Human intestinal mucus was isolated from 114 fecal samples collected from healthy infants and adults, and bifidobacteria, lactobacilli, and *A. muciniphila* were tested for their adhesive abilities to the intestinal mucus in vitro. In accordance with our previous study using the Caco-2 binding assay, LGG and Bb12 were used as positive controls and LA2 as a negative control [12].

**Figure 1:**
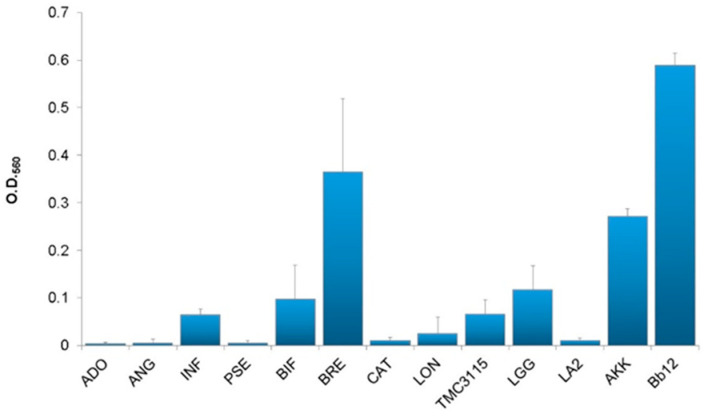
Adherence properties of bacterial strains to porcine mucin. Data are expressed as the mean ± SE. ADO: *Bifidobacterium adolescentis* JCM 2701 ^T^; ANG: *B. angulatum* ATTC 27678 ^T^; INF: *B. longum* subsp. *infantis* JCM 1222 ^T^; PSE: *B. pseudocatenulatum* JCM 1200 ^T^, BIF: *B. bifidum* JCM 1255 ^T^, BRE: *B. breve* JCM 1192 ^T^; CAT: *B. catenulatum* JCM 1194 ^T^; LON: *B. longum* subsp. *longum* JCM 1217 ^T^; AKK: *Akkermansia muciniphila* JCM 30893; LGG: *Lacticaseibacillus rhamnosus* GG; Bb12: *Bifidobacterium animalis* subsp. *lactis* Bb12; TMC3115: *B*. *bifidum* TMC3115; LA2: *L. rhamnosus* LA2.

**Figure 2:**
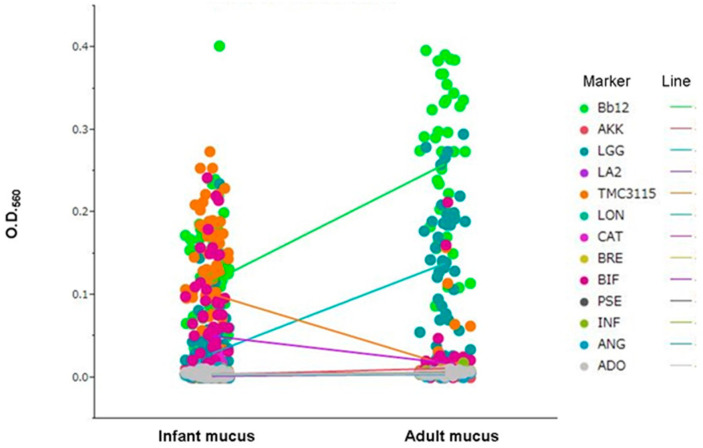
Adherence properties of bacterial strains to human mucus originating from infant and adult stools. Infant mucus was isolated from stools obtained from infants aged within 5 days old, 1 month old, 4 months old, 6 months old, 9 months old, 1 year old, 1 and a half years old, and 3 years old. Adult mucus was isolated from those aged 20–29, 30–39, 40–48, and 50–59 years old.

**Figure 3:**
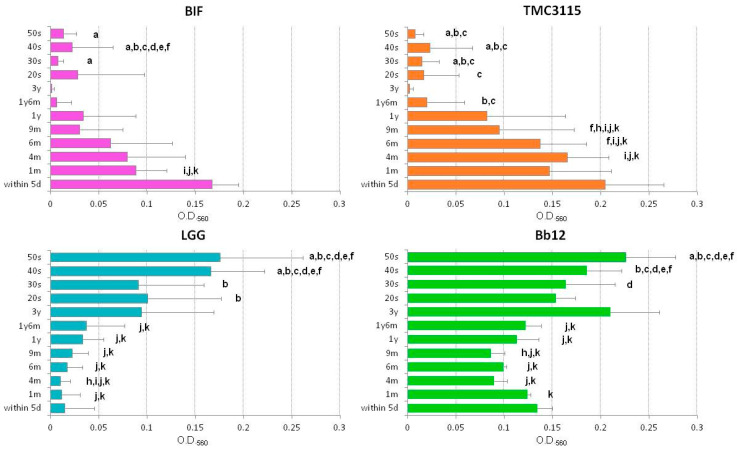
Age/generation-dependent adhesion properties of four highly human mucus adhesive strains. Data are expressed as the mean ± SE, and differences between means were tested for significance using a one-way ANOVA followed by the Kruskal–Wallis and Steel–Dwass tests. ^a,b,c,d,e,f,g,h,i,j,k^ Significantly different (*p* < 0.05) to adhesion to mucus from ^a^ 1m, ^b^ 4m, ^c^ 6m, ^d^ 9m, ^e^ 1y, ^f^ 1y6m, ^g^ 3y, ^h^ 20s, ^i^ 30s, ^j^ 40s, and ^k^ 50s.

In initial screening, 13 strains, including eight type strains of *Bifidobacterium*, TMC3115, LA2, LGG, AKK, and Bb12, were included to testing for their adhesive abilities. The adhesion properties of Bb12, BRE, and AKK to porcine stomach mucin were strong, whereas those of LGG, BIF, INF, and TMC3115 were moderate. Strong adhesion was not observed for the remaining six strains (Figure 1). The adhesion properties of these strains markedly changed when human mucus was applied to the assay. Four out of the 13 strains, including BIF, TMC3115, LGG, and Bb12, exhibited strong adhesion properties to human mucus in different age/generation-specific manners, while those of the remaining nine to any of the human mucus tested were weak (Figure 2). The adhesion of BIF and TMC3115 to neonatal mucus obtained from the feces of 1-month-old infants was significantly stronger than to those from adults in their 30s, 40s, and 50s (*p* < 0.05) (Figure 3). Furthermore, TMC3115 tended to have stronger adhesion to neonatal mucus from within 5 days old than to adult mucus (*p* < 0.2) and adhered more strongly to mucus from infants aged 4 and 6 months old than to adult mucus (*p* < 0.05). In addition, BIF and TMC3115 tended to adhere strongly to the mucus of infants aged 1, 4, and 6 months old than to that from infants aged 3 years old (*p* < 0.1), whereas their adherence to mucus from infants aged 4 and 6 months old was significantly stronger than to that from infants aged 1 year and 6 months old (*p* < 0.05). On the other hand, LGG and Bb12, well-known probiotic strains included as controls, exhibited different adhesion properties. LGG exhibited minor adhesion properties to neonatal and infant mucus (younger than 1 and half years old) but adhered well to adult mucus (Figure 3). LGG adhered significantly stronger to mucus originating from feces of adults in their 40 s and 50 s than to neonatal and infant mucus (1, 4, 6, and 9 months, 1 year, and 1 and half years) (Figure 3). Similar results were obtained for Bb12, both of which adhered well to mucus originating from the feces of adults in their 40s and 50s, while Bb12 showed stronger adhesion properties to infantile mucus than LGG. Distinct adhesion patterns were observed from these four highly human mucus adhesive strains to mucus originating from infants aged 1 and half years old and 3 years old.

**Figure 4:**
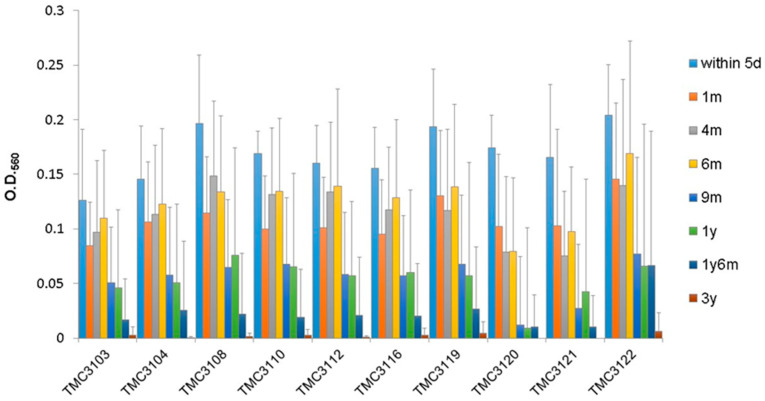
Adherence of ten additional strains of *Bifidobacterium bifidum* to human intestinal mucus.

Due to the unique adhesion properties found in the two *B. bifidum* strains, ten additional strains of *B. bifidum* were included in the adhesion assay. Similar adhesion properties were observed in all *B. bifidum* strains tested, and ten additional strains exhibited stronger adhesion abilities to mucus originating from infants younger than 1 year old (Figure 4). No strains strongly adhered to mucus originating from infants aged 3 years old.

## 4. Discussion

Exogenous bacteria, including probiotics, cannot easily adhere to gut mucus because of the presence of host gut microbiota. However, it is advantageous for demonstrating beneficial properties if probiotics could adhere to mucus layer. In fact, specific probiotics have been found as a part of the host gut microbiota [16], and an in vitro evaluation of bacterial adhesion to mucus provides a good model for studying the ability of probiotics to adhere to intestinal surfaces. The present study initially screened three strong and four moderate adherents to porcine gastric mucin among the 13 strains tested (Figure 1). Of these seven adherents, only four showed marked adhesion to human mucus with different age/generation-dependent adhesion properties (Figure 2), indicating that adhesion properties to porcine gastric mucin do not guarantee adhesion to human intestinal mucus. Former studies showed that *A. muciniphila* strongly adheres to the Caco-2 and HT-29 human colonic cell lines but not to human colonic mucus [17], however, age/generation-dependent adherence properties in *A. muciniphila* have not been studied. The present study clearly indicated that the organism adheres well to the porcine mucin but not to the human mucus irrespective of age/generation.

The strains, that did not strongly adhere to porcine mucin, also did not adhere to human mucus. These results suggest that porcine mucin is acceptable for the initial exclusion of non-adherent microbes to human mucus, but is not sufficient to screen probiotics exhibiting adhesion properties to human mucus. This would be due to differences in the chemical properties of mucus, particularly glycosylation patterns, between animals [18]. 

Age may also affect mucus properties. Glycoprotein compositions in the small intestine markedly differed between newborn rats (~24 h old) and adult rats (>2 months old) [19]. Specifically, levels of fucose, mannose, galactose, N-acetylgalactosamine, and sulphates were lower in newborn rats than in adult rats, while those of N-acetyglucosamine and N-acetylneuraminic acid were similar. Similar differences were observed between newborn (0 day) and mature (180 days) porcine colonic mucins, with older pigs containing higher levels of fucose and lower levels of proteins [20]. Potential differences in the chemical compositions of human mucus between ages/generations, which need to be confirmed in a future study, may affect the adhesion properties of gut microbes. The strains tested in the present study showed age/generation-dependent adhesion properties. Twelve strains of *B. bifidum,* including TMC3115, the type strain, and ten additional isolates from infant feces, showed strong adhesion properties to neonatal and infantile mucus, but not to adult mucus. To the best of our knowledge, this is the first study to demonstrate that *B. bifidum* compared to that of mucus from infants, particularly neonates, than that from adults, while the well-characterized probiotics LGG and Bb12 adhere more strongly to the mucus isolated from adults. A previous study isolated *B. bifidum* from infantile mucosal samples, suggesting that the microbe is a component of the mucosal microbiota of infants [21]. These findings suggest that a deeper symbiotic relationship exists between neonates and *B. bifidum*, which may contribute to a more detailed understanding of the ecological niche of *B. bifidum*, more so in infants than in adults, as described previously [22]. The present results also suggested that the health promoting effects of bifidobacteria may be evaluated based on the species level, but not the genus level; even bifidobacteria are generally regarded as beneficial intestinal microbes. Furthermore, *B. bifidum* strains did not strongly adhere to the intestinal mucus from infants aged 3 years old. This organism becomes less prevalent (~20%) 3 years after birth [23], which may be due to the poor adhesion properties of *B. bifidum* to intestinal mucus originating from infants older than 3 years old, as observed in the present study. A previous study reported that the phylogenetic composition of gut bacterial communities evolves towards an adult-like configuration within the first 3 years of life [24].

Specific strains of *B. bifidum,* for example, *B. bifidum* TMC3115, may alter host immunity in a different manner from other bifidobacteria, and modify the allergic responses of host animals [14,25,26]. Furthermore, *B. bifidum* was found to possess many extracellular glycosidases specified for degrading host-derived glycans, including HMOs, by which mono- and disaccharides are liberated from host glycans and HMOs and released into living environmental niches or culture medium in vitro [27,28]. Therefore, *B. bifidum* could support the growth of other bifidobacteria by sharing products derived from glycan degradation within the community. These findings suggest that *B. bifidum* plays a more important and unique role in maintaining host physiological homeostasis by the proliferation of beneficial gut microbes and the modulation of immunity through cooperation with gut microbes. *B. bifidum* PRL2010 is one of the well-studied strains of *B. bifidum* from the human intestine. This strain was found to possess the specific ability to adhere more strongly to human cell lines, such as Caco2 and HT29 monolayers, than other extensively characterized bifidobacterial probiotic strains, such as Bb12 [29]. The production of sortase-dependent pili was previously reported to be crucial for the strain to adhere to human enterocytes and modulate microbe-host crosstalk [21,29]. Recent studies also indicated that pilus production was enhanced when *B. bifidum* PRL2010 cells were located in their natural ecological niche, i.e., the human gut, or when this habitat was simulated under in vitro conditions, e.g., by the incubation of PRL2010 in the presence of complex carbohydrates commonly found in the human large intestine [4]. A genomic analysis indicated that *B. bifidum* TMC3115 also possesses the gene cluster involved in sortase-dependent pilus production in the genome [27]. These findings suggest that the unique adhesion traits found in *B. bifidum* strains to neonatal mucus are the result, at least partly, of pilus production by these bacteria. Another possibility is the presence of the cell-surface moonlight proteins of *B. bifidum*. A previous study reported that an extracellular sialidase of the organism (SiaBb2), which cleaves sialyl-human milk oligosaccharides and mucin glycans, mediated the adhesion of these bacteria to human epithelial cells and porcine mucin [30]. Moreover, SiaBb2 enhanced *B. bifidum* adhesion to mucosal surfaces via specific interactions with the 2, 6 linkage of sialyloligosaccharide and blood type A antigen on mucin carbohydrates. Future studies are needed to identify the key factors influencing the age/generation-dependent adhesion properties of the organism. 

In conclusion, lactobacilli and bifidobacteria exhibited age/generation-dependent adhesion properties to human mucus. *B. bifidum* strains adhered well to neonatal and infantile mucus, and LGG and Bb12 to adult mucus. These results suggest that target age groups need to be considered when developing probiotics. Porcine gastric mucin is not a suitable tool for the selection of probiotics adhering to human mucus.

## Data Availability

Not applicable.
